# Cell Survival and Apoptosis Signaling as Therapeutic Target for Cancer: Marine Bioactive Compounds

**DOI:** 10.3390/ijms14022334

**Published:** 2013-01-24

**Authors:** Senthilkumar Kalimuthu, Kim Se-Kwon

**Affiliations:** Marine Bioprocess Research Center, Department of Chemistry, Pukyong National University, Busan 608-737, Korea; E-Mail: senthilbhus@gmail.com

**Keywords:** apoptosis, cell survival, AKT, Bax, marine compounds

## Abstract

Inhibition of apoptosis leads to activation of cell survival factors (e.g., AKT) causes continuous cell proliferation in cancer. Apoptosis, the major form of cellular suicide, is central to various physiological processes and the maintenance of homeostasis in multicellular organisms. A number of discoveries have clarified the molecular mechanism of apoptosis, thus clarifying the link between apoptosis and cell survival factors, which has a therapeutic outcome. Induction of apoptosis and inhibition of cell survival by anticancer agents has been shown to correlate with tumor response. Cellular damage induces growth arrest and tumor suppression by inducing apoptosis, necrosis and senescence; the mechanism of cell death depends on the magnitude of DNA damage following exposure to various anticancer agents. Apoptosis is mainly regulated by cell survival and proliferating signaling molecules. As a new therapeutic strategy, alternative types of cell death might be exploited to control and eradicate cancer cells. This review discusses the signaling of apoptosis and cell survival, as well as the potential contribution of marine bioactive compounds, suggesting that new therapeutic strategies might follow.

## 1. Introduction

Apoptosis is critically important for the survival of multicellular organisms [1]. Apoptosis triggered by exogenous and endogenous stimuli, such as ultraviolet radiation, oxidative stress and genotoxic chemicals, is a crucial phenomenon within biological systems. Cell death could be caused by necrosis and apoptosis. The extrinsic and intrinsic pathways represent the two major well-studied apoptotic processes [2,3]. The extrinsic pathway mediated by the sub group of Tumor Necrosis Factor receptors (TNF-R) super family that includes TNF-R, Fas and Tumor necrosis factor receptor is related to apoptosis inducing ligand (TRAIL). Activation of these so-called death receptors leads to the recruitment and activation of initiator caspases, such as caspases 8 and 10. The process involves the formation and activation of complexes, such as the death inducing signaling complex (DISC). This leads to the activation of an effector caspase, typically caspase 3. The active caspase 3 responsible for the cleavage of number of death substrates leads to the well-known characteristic hallmarks of apoptotic cell, including DNA fragmentation, nuclear fragmentation, membrane blebbing and other morphological and biochemical changes. More evidences suggest that greater complexity and diversity in the extrinsic pathways also involves the cross-activation of other apoptotic pathways, such as the intrinsic apoptotic, as well as necrotic sub pathways [2,4].

The mitochondrial pathway of apoptosis is initiated by a variety of upstream stimuli and tightly regulated by various factors, including pro and anti apoptotic proteins of the Bcl-2 family, as well as the phosphatidyl inositol 3-kinase (PI3K)/AKT/mammalian target of rapamycin (mTOR) pathway [5,6]. The PI3K/AKT/mTOR signaling cascade belongs to the critical survival programs that are typically over activated in human cancers, promoting cell survival by inhibiting the apoptosis [7]. The PI3K signaling network diversifies into many distinct downstream branches, one of which leads to the activation of mTOR [8]. In addition, intricate interactions between distinct kinase of survival networks had been described. Since small-molecule inhibitors that block PI3K/AKT/mTOR signaling are currently undergoing clinical evaluation in early trials, there is much interest to understand how these inhibitors interfere with intracellular signaling pathways, for example, mitochondria-mediated apoptosis. There are number of reviews evidencing apoptosis, but this paper gives a short overview on PI3K/AKT regulation and an apoptosis discussion about some marine bioactive compounds on these aspects.

## 2. Cell Survival

### 2.1. Survival Signaling via PI3K/AKT

AKT plays an important role in cell survival [9]. The PI3K/AKT pathway represents a key signal transduction pathway that mediates cell growth and blocks apoptosis [8]. Increased activation of this survival cascade is a characteristic feature of a large variety of human malignancies and has been associated with carcinogenesis [5]. Growth factors binding to their corresponding receptors results in the activation of this survival cascade in order to regulate the intracellular signaling that supports proliferation and survival. Activation of growth factor receptors results in the phosphorylation of receptor tyrosine kinases that resides within the plasma membrane. This, in turn, activates the whole cascade of the PI3K/AKT pathway. This signaling is antagonized by the tumor suppressor gene phosphatase and tensin homologue deleted on chromosome 10 (PTEN), which acts as both a lipid and a protein phosphatase [10]. PTEN dephosphorylates PIP3 in to PIP2, thereby negatively regulating PI3K/AKT signal transduction.

### 2.2. Role of AKT in Cell Survival

**Figure 1:**
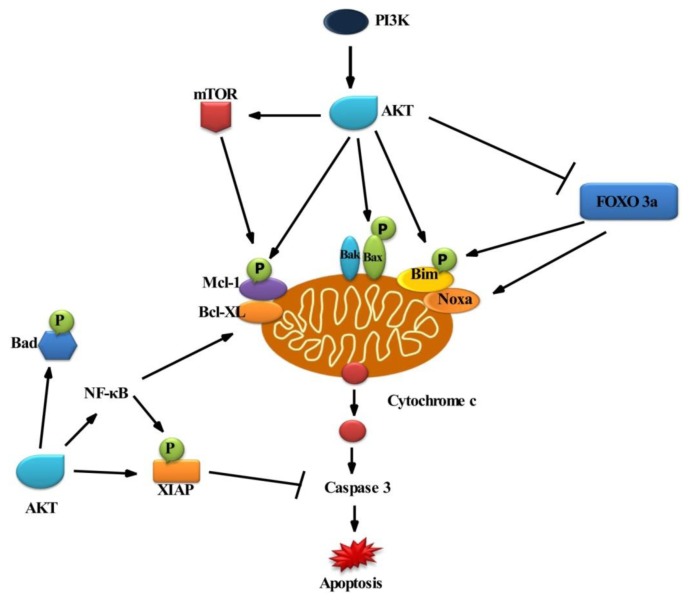
Scheme of PI3K/AKT mediated antiapoptotic regulations at the mitochondria. AKT phosphorylated by PI3K activation. AKT phosphorylates and inhibits Bax and Bad (both are proapoptotic protein). AKT activates mTOR, which in turn phosphorylates and activates antiapoptotic protein MCL-1 (induced myeloid leukemia cell differentiation protein). AKT also activates NF-κB (nuclear factor kappa B), thus resulting in transcription of pro-survival gene Bcl-XL (B-cell lymphoma-extra large). AKT phosphorylates and inhibits proapoptotic protein Bax. NF-κB phosphorylates the X-linked inhibitor of apoptosis protein (XIAP), then binds to and inhibits caspases. Bim (Bcl-2 interacting mediator of cell death) and Noxa are the only proapoptotic BH-3 protein inhibited by FOXO3 protein (Forkhead box O3), phosphorylated by AKT in the PI3K signaling pathway.

AKT acts as a cell survival factor by regulating apoptotic signal transduction directly or indirectly [8]. AKT has been reported to directly interfere with cell death pathways by phosphorylating key apoptosis-regulatory proteins, which, in turn, results in a shift within the ratio of pro- and anti-apoptotic proteins towards the inhibition of cell death (Figure 1).

**Figure 2:**
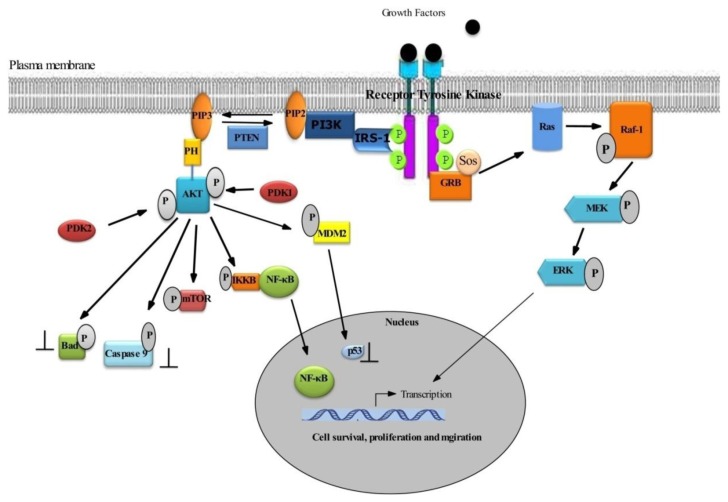
Scheme of survival signaling cascade by receptor tyrosine kinases via PI3K/AKT mediated survival. The binding of a ligand (Growth Factor) to its receptor triggers intrinsic tyrosine kinase phosphorylation, subsequently activating IRS and PI3K. Activated PI3K generates phosphatidylinositol-3,4,5-triphosphate, which recruits phosphatidylinositol-dependent kinase (PDK) and AKT serine/threonine kinase at the plasma membrane, resulting in phosphorylation of AKT. After phosphorylation, activated AKT inactivates other apoptogenic factors, Bad (a pro-apoptotic protein, which in its non-phosphorylated state, promotes apoptosis) and caspase 9. AKT also activates mTOR by phosphorylation. The transcription factor NF-κB can lead to transactivation of a wide range of antiapoptotic NF-κB target genes (e.g., Bcl-XL and Bcl-2). AKT also phosphorylates MDM2 (murine double minute protein), which in turn inhibits p53 action. On the other hand, cell survival and proliferative signals are mediated by the Ras/Raf pathway (Figure 2). Receptor tyrosine kinase phosphorylation also activates the GRB/SOS2 (Growth factor receptor-bound protein 2/Son of Sevenless, adaptor proteins), which in turn activates Ras (Rous sarcoma protein). Then, Ras activates Raf-1(Receptor activated factor-1) kinase, which subsequently activates the MEK and ERK, thereby regulating the survival, proliferation, migration and invasion of cancer.

Abundant evidence implicates the PI3K/AKT pathway in the development and progression of multiple tumors. AKT is activated in response to many growth factors, hormones and cytokines. Binding the growth factor to its receptor triggers intrinsic tyrosine kinase phosphorylation, subsequently activating IRS (insulin receptor substrate) and PI3K (Figure 2).

Activated PI3K generates phosphatidylinositol-3,4,5-triphosphate, which recruits phosphatidylinositol-dependent kinase (PDK) and AKT serine/threonine kinase at the plasma membrane, resulting in phosphorylation of AKT. The central regulatory mechanism is cell survival. BAD is a pro-apoptotic protein of the Bcl-2 family. AKT could phosphorylate BAD on Ser136, which makes BAD dissociate from the Bcl-2/Bcl-X complex and lose the pro-apoptotic function. Akt could also activate NF-κB via regulating IκB kinase (IKK), thus resulting in transcription of pro-survival genes. Omi/high temperature requirement proteinA2 (HtrA2), a mitochondrial serine protease, is released from the mitochondria to the cytosol during apoptosis. AKT phosphorylates Omi/HtrA2, thereby inhibiting apoptosis [7]. In addition, the multi-domain Bcl-2 protein Bax has been shown to be phosphorylated by AKT in a dependent manner at serine residue 184 [11,12]. AKT mediated phosphorylation of Bax leads to changes in the conformation of Bax and blocks its activation. Besides, Bim (Bcl-2 interacting mediator of cell death) is a BH3 (Bcl-2 homology domain) protein phosphorylated at serine residue, *i.e.*, Ser87 [13], by AKT. While the phosphorylation of proapoptotic Bcl-2 proteins, Bax and Bim, reduces their proapoptotic potential, AKT mediated phosphorylation of some antiapoptotic factors, such as XIAP (X-linked inhibitor of apoptosis protein) and Mcl-1 (induced myeloid leukemia cell differentiation protein), decrease their antiapoptotic properties by decreasing their protein stability. Accordingly, phosphorylation of XIAP and Mcl-1 by AKT promotes the degradation of these proteins via the proteasomal machinery, resulting in reduction of XIAP and Mcl-1protein expression [14,15].

Apart from the direct interference of AKT with cell death signaling pathways via phosphorylation of key signal transduction molecules, AKT has also been reported to interfere with cell death programs indirectly via the phosphorylation of transcription factors. This mechanism applies to the transcription factor FOXO3 (Forkhead box O3). It belongs to the O subclass of the forkhead family of transcription factors, which are characterized by a distinct forkhead DNA-binding domain. FOXO3 transcription factors, such as FOXO1, FOXO3a, FOXO4 and FOXO6, are involved in the apoptotic mechanism. FOXO3 upregulates *Bim* and *Noxa* gene expression [16,17]. FOXO3 action is inhibited by phosphorylation of AKT in the PI3K signaling pathway (Figure 1). AKT mediated activation of the transcription factor NF-κB transactivates wide range of antiapoptotic NF-κB target genes, including inhibitor of apoptosis (IAP) proteins, Bcl-XL and Bcl-2 [18]. PI3K/AKT signaling represents a key regulatory mechanism to control the activity of pro- and anti-apoptotic Bcl-2 family proteins. Accordingly, small-molecule inhibitors of PI3K/AKT signaling reported that the balance between pro- and anti-apoptotic proteins towards apoptosis decrease the expression of Mcl-1 and increase phosphorylation of Bim [19–22]. Phorbol-12-myristate-13-acetate-induced protein 1 is also known as Noxa, a pro-apoptotic member of the Bcl-2 protein family. Increased expression of Noxa may promote mitochondrial apoptosis by directly binding to Mcl-1, thereby antagonizing the antiapoptotic function of Mcl-1 [23].

## 3. Apoptosis

### 3.1. Mechanism of Apoptosis

The process of programmed cell death, or apoptosis, is generally characterized by distinct morphological characteristics and energy-dependent biochemical mechanisms. Impairment of this native defense mechanism promotes aberrant cellular proliferation and the accumulation of genetic defects, ultimately resulting in tumorigenesis [24]. There are two pathways triggered during apoptosis: the intrinsic (mitochondrial) and extrinsic (death receptor) mediated pathway [25]. This apoptotic pathway is centrally regulated by upstream activators. Among these are PI3K/AKT/mTOR signaling, proapoptotic proteins of the Bcl-2 family, cellular stress stimuli and hypoxia [6]. The intrinsic pathway of apoptosis, permeabilization of outer mitochondrial membrane, is associated with the release of mitochondrial proteins from the intermembrane space into the cytosol, such as cytochrome c, second mitochondrial activator of caspases (Smac) and apoptosis inducing factor (AIF). Cytochrome c promotes the aggregation of caspase-9 together with Apaf-1 in the cytosol to form a multi-protein complex called an apoptosome, which results in caspase-9 activation. The release of Smac from the mitochondrial intermembrane space into the cytosol promotes apoptosis by binding to the inhibitor of apoptosis (IAP) proteins [26]. p53 is considered to be “a cellular gatekeeper for growth and division” by controlling critical cell cycle checkpoints [27]. The p53 mediates apoptosis through activation of APO-1/Fas and other death receptors and/or up- and down-regulation of Bax and Bcl-2, respectively [28,29]. p53 is activated by various stress conditions, including radiation. Increased production of reactive oxygen species (ROS) under stress, which activates stress responsive pathways (p38MAPK), thereby promotes apoptosis [30–32].

### 3.2. Intrinsic Pathway

The intrinsic apoptotic pathway is characterized by membrane permeability that causes mitochondrial swelling, rupture of the outer membrane and release of proapoptotic factors from the intermembranous space. This is achieved by (1) an opening of the permeability transition pore and (2) an increase of the Bax/Bcl-2 ratio 3. The intrinsic signaling pathways mediating apoptosis involves the absence of certain growth factors, hormones and cytokines. Apoptotic stimuli include radiation, drugs, toxins, hypoxia, hyperthermia, viral infections and free radicals. All of these stimuli cause changes in the inner mitochondrial membrane that result in an opening of the mitochondrial permeability transition (MPT) pore and release of two main groups of normally sequestered pro-apoptotic proteins from the intermembrane space into cytosol [33]. The first group consists of cytochrome c, Smac/DIABLO and the serine protease, HtrA2/Omi [34–36]. Cytochrome c binds and activates Apaf-1, forming an “apoptosome” [37,38], adenosine tri phosphate (ATP), also required for the activation of apoptosome complex, which in turn activates procaspase-9 into active caspase-9. DIABLO is also known as SMAC (second mitochondria-derived activator of caspases), and HtrA2/Omi are reported to promote apoptosis by inhibiting IAP (inhibitors of apoptosis proteins) activity [37,39].

The second group of pro-apoptotic proteins, AIF (apoptosis-inducing factor), endonuclease G and CAD (caspase-activated DNase), are released from the mitochondria during apoptosis. AIF, a mitochondrion-localized flavoprotein, triggers chromatin condensation and DNA degradation during apoptosis. AIF translocates to the nucleus and causes DNA fragmentation into 50–300 kb pieces and condensation of peripheral nuclear chromatin [40,41]. This early form of nuclear condensation is referred to as “stage I” condensation [42]. Endonuclease G is a mitochondrial enzyme that also translocates to the nucleus, where it cleaves nuclear chromatin to produce oligonucleosomal DNA fragments [43]. AIF and endonuclease G both function in a caspase-independent manner. CAD is an endonuclease released from the mitochondria and translocates to the nucleus, causing oligonucleosomal DNA fragmentation [44]. This later and more pronounced chromatin condensation is referred to as “stage II” condensation [42]. The control and regulation of mitochondrial apoptotic events occur through members of the Bcl-2 family of proteins [45]. The tumor suppressor protein p53 also plays a critical role in regulation of the Bcl-2 family of proteins [46].

The discovery of Bcl-2 did not drive cell proliferation, as for previously characterized oncogenes, but promotes cell survival in tumorigenesis [47,48]. The Bcl-2 gene was originally identified at the chromosomal breakpoint of the translocation of chromosome 18 to 14 in follicular non-Hodgkin lymphoma (NHL) [49]. Increased expression of Bcl-2 causes resistance to chemotherapeutic drugs and radiation therapy, while decreasing Bcl-2 expression may promote apoptotic responses to anticancer drugs [50,51]. The Bcl-2 family includes proapoptotic members, such as Bax, Bak, Bad, Bcl-Xs, Bid, Bik and Bim and antiapoptotic members, such as Bcl-2, Bcl-XL, Bcl-W, Bfl-1 and Mcl-1 [52,53]. Antiapoptotic Bcl-2 members act as repressors of apoptosis by blocking the release of cytochrome c, whereas proapoptotic members act as promoters [50]. Following a death signal, proapoptotic proteins undergo post translational modifications that include dephosphorylation and cleavage, resulting in their activation and translocation to the mitochondria, leading to apoptosis [54]. BH3-only molecules require multi-domain BH3 proteins (Bax, Bak) to exert their intrinsic proapoptotic activity [47,54,55].

In response to apoptotic stimuli, cytochrome c, released into the cytosol, interacts with Apaf-1, leading to the activation of caspase-9 proenzymes [56,57]. Active caspase-9 then activates caspase-3, which subsequently activates the rest of the caspase cascade and leads to apoptosis. Activated caspases lead to the cleavage of nuclear lamin and breakdown of the nucleus through caspase-3 [58].

### 3.3. Death Receptor Pathway

Death receptors are cell surface receptors that transmit apoptosis signals initiated by their specific “death ligands”. The cell surface death receptors belong to the superfamily of tumor necrosis factor receptors (TNF-R) and are activated by TNF family ligands. This pathway comprises several protein members, including the death receptors, the membrane-bound FasL, the Fas complexes, the Fas-associated death domain and caspases 8 and 10, which ultimately activate the rest of the downstream caspases, leading to apoptosis. Activation of the extrinsic pathway is initiated with the ligation of cell surface receptors, called death receptors (DRs). Fas is a member of the tumor necrosis factor receptor superfamily and is also called Apo-1 or CD95. Other TNF receptors include TNF R1, DR3 (Apo 2), DR4 (tumor necrosis factor related apoptosis-inducing ligand receptor 1 (TRAIL R1), DR5 (TRAIL R2) and DR6 [59]. Fas signaling plays an important role in immune surveillance of transformed or virus infected cells and in the removal of self reactive lymphocytes. Therefore, defects in this pathway have been implicated in many malignancies and autoimmune diseases [60,61].

The Fas ligand and Fas system is mainly recognized for its death-related functions, but it is also involved in several proliferative and inflammatory signaling pathways that are not well defined [62]. Fas ligand (FasL or CD95L) belongs to the tumor necrosis factor (TNF) family. It binds with its receptor to form Fas death-inducing signaling complex (DISC), contains the adaptor protein Fas-associated death domain protein and caspases 8 and 10 and leads to activation of caspase 8, which in turn activates the rest of the downstream caspases. In some cells, the activation of caspase 8 may be the only requirement to execute death, while in other cell types, caspase 8 interacts with the intrinsic apoptotic pathway by cleaving Bid (a proapoptotic member of the Bcl-2 family), activating the release of cytochrome c [63].

Alterations in the extrinsic pathway lead to malignant transformation, as mutations or deletions of the *Fas* gene has been found in some hematologic malignancies [61,64]. Regulators of the pathway include transcription factor NF-κB and activating protein 1 that regulate the *FasL* gene, because it is a transcriptionally inactive gene [63]. The extrinsic pathway of apoptosis is abrogated through several mechanisms, including the upregulation of the inhibitors of apoptosis proteins, such as cIAP or XIAP. Smac/DIABLO, a mitochondrial protein and negative regulator of XIAP, enhances apoptosis by binding with XIAP, thereby preventing binding to caspases. Other inhibitors of the pathway include FAP-1 (Fibroblast activation protein), Fas-associated-death-domain-protein, like interleukin-1, converts enzyme-like inhibitory proteins and the soluble decoy receptors, such as DcR3, TRAIL R-3/DcR1 and TRAIL R-4/ DcR2. These decoy receptors are antagonized by the stimulation of Fas ligand [65,66].

### 3.4. Final Pathway

The final pathway leads to activation of a family of cysteine aspartyl proteases (caspases) [67]. Caspases are a family of cysteine proteases, which contain cysteine residue at their active site and cleave their substrate at a position next to aspartate residue. The entire group of mammalian caspases is divided into three different groups on the basis of their prodomains and specific function in different pathways, including inflammatory, development and apoptotic pathways [48]. Caspases, totaling 14 family members, are synthesized as inactive zymogens, which have been proteolytically cleaved at two (or three in some cases) aspartate residues to generate the active mature enzyme. The generations of active caspases interact with specific adapter molecules to facilitate their own auto processing. Active initiator caspases, in turn, cleave and activate the downstream “executioner” caspases. These then cleave their target substrates to orchestrate the proteolytic dismantling of the cell [68,69]. Not all caspases are involved in apoptosis; caspases-3, -6, -7, -8 and -9 [70] are well described. The intrinsic and extrinsic apoptotic pathways converge to caspase-3, which cleaves the inhibitor of the caspase-activated deoxyribonuclease, and the caspase-activated deoxyribonuclease becomes active, leading to nuclear apoptosis. The downstream caspases induce cleavage of protein kinases, cytoskeletal proteins, DNA repair proteins, inhibitory subunits of endonuclease and, finally, destruction of “housekeeping” cellular functions. Caspases also affect cytoskeletal structure, cell cycle regulation and signaling pathways, ultimately leading to the morphologic manifestations of apoptosis, such as DNA condensation and fragmentation and membrane blebbing [71]. The intrinsic pathways are triggered by various extracellular and intracellular stresses, such as growth factor withdrawal and hypoxia; DNA damage and oncogene induction signals that are transduced in response to these stresses convert mainly on mitochondria. A series of biochemical events induced during apoptosis results in the permeabilization of the outer mitochondrial membrane, the release of cytochrome c and other proapoptotic molecules, the formation of apoptotic protease activating factor 1, caspase-9 and caspase-3 activation.

### 3.5. Biochemical Characteristics of Apoptosis

Apoptotic cells exhibit several biochemical modifications, such as protein cleavage, protein cross-linking, DNA breakdown and phagocytic recognition that together result in the distinctive structural pathology described previously [72]. Caspases are widely expressed as inactive proenzyme forms in most cells and, once activated, can often activate other procaspases, allowing initiation of the protease cascade. With this proteolytic cascade, one caspase can activate other caspases, amplifying the apoptotic signaling pathway and, thus, leading to rapid cell death. Caspases are able to cleave proteins at aspartic acid residues, although different caspases have different specificities involving recognition of neighboring amino acids. Once caspases are initially activated, there seems to be an irreversible commitment towards cell death. Caspases are identified and broadly categorized into initiators (caspase-2,-8,-9,-10), effectors or executioners (caspase-3,-6,-7) and inflammatory caspases (caspase-1,-4,-5) [73,74].

The other caspases that have been identified include caspase-11, which has been reported to regulate apoptosis and cytokine maturation during septic shock, caspase-12, which mediates endoplasmic-specific apoptosis and cytotoxicity by amyloid-β, caspase-13, which has been suggested in bovine gene, and caspase-14, which is highly expressed in embryonic tissues, but not in adult tissues [75–78]. Extensive protein cross-linking is another characteristic of apoptotic cells achieved through the expression and activation of tissue transglutaminase [79]. DNA breakdown by Ca^2+^ and Mg^2+^ dependent endonucleases also occur, resulting in DNA fragments of 180 to 200 base pairs. With the terminal transferase mediated DNA nick end labeling (TUNEL) assay, cells containing DNA strand breaks become visualized by fluorescent microscope [80]. A characteristic “DNA ladder” was visualized by agarose gel electrophoresis with ethidium bromide staining in ultraviolet illumination. Another biochemical feature is the expression of cell surface markers that result in the early phagocytic recognition of apoptotic cells by adjacent cells, permitting quick phagocytosis with minimal compromise to the surrounding tissue. It is achieved by the movement of the normal inward facing phosphatidylserine of the cell’s lipid bilayer to expression on the outer layers of the plasma membrane [81]. Although externalization of phosphatidylserine is a well known recognition ligand for phagocytes on the surface of the apoptotic cell, studies showed that other proteins are also exposed on the cell surface during apoptotic cell clearance. These include Annexin I and calreticulin. Annexin V is a recombinant phosphatidylserine-binding protein that interacts strongly and specifically with phosphatidylserine residues and could be used for the detection of apoptosis [82]. Calreticulins are protein that binds to an LDL-receptor related protein on the engulfing cell and has suggested cooperation with phosphatidylserine as a recognition signal during apoptosis [83].

## 4. Chemoprevention

Chemoprevention, a relatively new and promising strategy to prevent cancer, is defined as the use of natural dietary compounds or synthetic substances to block, inhibit, reverse or retard the process of carcinogenesis [84]. Cancer development, a dynamic and long-term process, involves many complex factors with a stepwise progression that ultimately leads to metastasis, an uncontrolled spreading and growth of cancerous cells throughout the body. Large-scale clinical studies have demonstrated treatment for breast cancer with the efficacy of using tamoxifen, raloxifene, both estrogen receptor antagonists and fenretinide, a synthetic retinoid [84–86]. Chemotherapy aims to kill cancer cells in the hope of preventing further cancer progression. Chemoprevention, on the other hand, involves administering non-toxic agents to individuals who may be at an increased risk for cancer. Moreover, surgical and traditional therapeutic approaches (chemotherapy and radiation) are, at present, unable to control most cancer types. Thus, the development of new chemopreventive strategies is required [87]. Chemopreventive compounds are classified into two major groups: (1) blocking agents, which prevent carcinogens from reaching or reacting with critical target sites and (2) suppressing agents, which stop the evolution of pre-neoplastic processes. Given that the initiation and progression phases are relatively transient and irreversible events, it seems that chemopreventive agents should intervene at the prodromal promotion phase. Three decades of research suggest that chemoprevention is a promising strategy to reduce the incidence of cancer, both in well-defined high-risk groups and in the general population [88–90].

Focus has been on the molecular basis of chemopreventive potential of natural compounds, with special emphasis on their effects in cellular signaling molecules as a target. To study the biological effects of phytochemicals at the cellular level provides the molecular basis for their function and helps to establish more potent chemopreventive agents. Many studies have been carried out to find cancer chemotherapeutic agents from edible and natural resources, such as fruits, vegetables and terrestrial plants [91,92]. The marine environment represents a relatively untapped source of functional ingredients. The marine derived bioactive compounds also have important sources for dietary supplements, and a number of them are potentially active.

### The Potential Effect of Marine Bioactive Compounds on Cancer Cell Survival and Apoptosis

Nature has been an important source of novel anti-cancer drug leads over the past 25 years [93], with increasing numbers of new compounds sourced from the marine environment [94]. The chemical and biological diversity of the marine environments are an immeasurable and extraordinary resource for the discovery of new anticancer drugs. Approximately 22,000 natural products of marine origin were discovered so far, whereas 131,000 terrestrial natural products exist [95]. Natural compounds remain a high output source of promising chemotherapeutic or chemopreventive agents in current cancer research [96,97].

Marine organisms, including sponges [98], sponge-microbe symbiotic association [99], gorgonian [100], actinomycetes [101] and soft coral [102], have been widely explored for potential anticancer agents. The effects of marine nutraceuticals on apoptotic pathways, signaling pathways and/or different targets in cancer mean that they could be helpful starting points in the design and development of novel cancer preventive agents. Of great importance, aberrant NF-κB regulation and AKT activation has been observed in many cancers. To prevent the development and progression of cancers, the strategy should target the cell signaling pathways in cancer. Aberrant regulation of NF-κB and the signaling pathways that control its activity are involved in cancer development and progression, as well as in drug resistance, especially during chemotherapy and radiotherapy [103]. Blocking NF-κB could cause tumor cells to cease proliferation or become more sensitive to the action of antitumor agents [104]. Changes in Akt activator expression has been observed in human precancerous tissues that might be targeted for chemoprevention [105]. Thus far, several chemopreventive agents have shown their various activities in the inhibition of carcinogenesis through the regulation of major cell signaling pathways, such as AKT and NF-κB. Therefore, those are the subject of intense study. There are a number of marine derived compounds related to research topic progress worldwide. Here is a list of some marine derived bioactive compounds that show evidence that they regulate cell survival and apoptosis mechanisms (Table 1).

PI3K/AKT pathway transmits antiapoptotic survival signals, and their inhibition by marine compounds could be a potential therapeutic value. Agents capable of suppressing AKT and/or NF-κB activation have therapeutic promise and the potential to inhibit carcinogenesis. The pathway-based phosphor profiling approach identifies and quantifies clinically relevant, drug-specific biomarkers for PI3K pathway inhibitors that target AKT, phosphoinositide dependent kinase 1 (PDK1), PI3K and mammalian target of rapamycin (mTOR) [106]. Arenamides (A-C) from the fermentation broth of a marine bacterial strain *Salinispora arenicola* blocked TNF-induced activation of NF-κB in a dose- and time-dependent manner [107]. Even marine derived sediments are rich in bioactive substances, which may interact with NF-κB, thereby inducing apoptosis in cancer cells.

Heteronemin, a marine sesterterpene isolated from the sponge *Hyrtios* sp., inhibits NF-κB activation and activates both initiator caspases -8 and -9, which are implicated in the extrinsic and intrinsic apoptotic pathway, respectively, in chronic myelogenous leukemia cells [108]. Tyrindoleninone and 6-bromoisatin are indole derivatives from marine mollusc *Dicathais orbita* and induce apoptosis in female reproductive cancer cell lines for ovary, granulosa and choriocarcinoma (OVCAR-3, KGN and Jar), respectively. Further studies, including investigation of initiator caspase 8 and 9, could help discriminate between the extrinsic and intrinsic pathway for the induction of apoptosis by these compounds [109]. The ester-substituted sesquiterpenoid cryptosphaerolide is isolated from the marine-derived ascomycete fungal strain CNL-523 (*Cryptosphaeria* sp.). Cryptosphaerolide was found to be an inhibitor of the protein Mcl-1(induced myeloid leukemia cell differentiation protein), a cancer drug target involved in apoptosis in HCT-116 human colon carcinoma cell line, indicating that the compound may be of value in exploring the Mcl-1 as a target for cancer chemotherapy [110]. Mcl-1, an anti-apoptotic member of the Bcl-2 family, sequesters Bak on the outer mitochondrial membrane, thereby preventing Bak oligomerization. Inhibition of Mcl-1promote cytochrome release, thereby, induces apoptosis.

Makaluvamine A is a pyrroloquinoline, principally isolated from the sponge *Zyzzya fuliginosa*, and is known to have potent anticancer activity in HCT-116 cells [111]. Ascididemin (ASC), an aromatic alkaloid isolated from the Mediterranean ascidian *Cystodytes dellechiajei* [112], is a strong inducer of apoptosis in HL-60 and P388 leukemia cells [113]. Another alkaloid, Lamellarin D (LAM-D), initially isolated from a prosobranch mollusc of the genus *Lamellaria*, exhibits cytotoxicity against many different tumors. LAM-D potently stabilizes topoisomerase I DNA covalent complexes to promote the formation of DNA single strand breaks. LAM-D also promotes nuclear apoptosis in leukemia cells via the intrinsic apoptotic pathway. Activation of Bax decreases the expression of antiapoptotic proteins Bcl-2 in association with activation of caspase-9 and caspase-3 [114,115]. Spongistatin 1, a macrocyclic lactone isolated from the marine sponges *Spirastrella spinispirulifera* and *Hyrtios erecta* induces apoptosis by interacting with the caspase-dependent pathway by the release of cytochrome c, Smac/DIABLO and Omi/HtrA2 from the mitochondria to the cytosol, leading to apoptosis in Jurkat cells [116]. Streptochlorin is a small molecule isolated from *Streptomyces* sp. and exhibits selective cytotoxicity against several cancer cell lines [117,118]. Streptochlorin induces apoptosis in human leukemic U937 cancer cells by a dose- and time-dependent manner by modulation of the Fas/Fas ligand (FasL) system, downregulating anti-apoptotic Bcl-2 expression and upregulating pro-apoptotic protein Bax [119].

**Figure 4:**
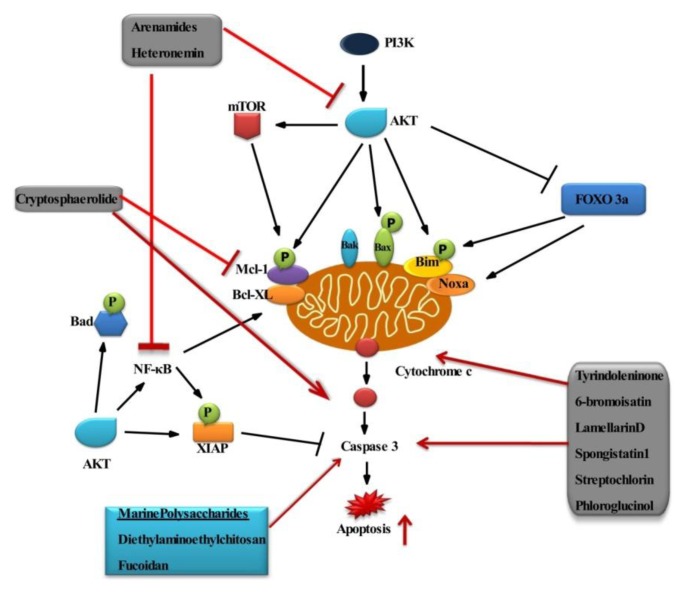
Interaction of marine compounds on survival and apoptotic signaling molecules. Regulation of this pathway with marine compounds; arrow indicates activation and blocked arrow indicates inhibition of the molecules.

Seaweeds belong to a group of plants known as algae. Seaweeds are classified as Rhodophyta (red algae), Phaeophyta (brown algae) or Chlorophyta (green algae), depending on their nutrient, pigment and chemical composition. Like other plants, seaweed contains various inorganic and organic substances, which could benefit human health [120]. Seaweed has a variety of chemoprotective compounds, such as flavonoids and other phenolic compounds [121]. The brown algae *Ecklonia Cava* (Laminariaceae), distributed abundantly in the sea all over the world, has been used as a seasonal vegetable in coastal areas. This is one of the seaweeds growing at a water depth of 2–25 m in the sublittoral zone along the coast of Korea [122]. Phloroglucinol, derived from *Ecklonia Cava*, causes cancer inhibition in MCF-7 human breast cancer cells and induces apoptosis [123]. Marine bioactive compounds interactions on the signaling molecules are depicted in the Figure 4.

Marine derived polysaccharides from microorganisms and seaweed have potential effects in the biomedical and pharmaceutical fields. Chitosan is produced commercially by deacetylation of chitin, which has the structural element in the exoskeleton of crustaceans (such as crabs and shrimp) and cell walls of fungi. Chitosan, a copolymer consisting of (1→4)-2-acetamido-d-glucose and (1→4)-2-amino-d-glucose units, is derived from chitin by deacetylation. Chitosan is the second most abundant polysaccharide in nature, and its production is low cost and ecologically appealing. Due to its biocompatibility and less toxic nature, it is being developed as a new physiologically bioactive material, because it possesses various biological activities, such as antioxidant and antitumor activity [124]. Diethylaminoethyl chitosan induces apoptosis in HeLa cells via activation of caspase-3 and p53 expression [125]. Fucoidan is the collective name for algal sulfated polysaccharides extracted from the brown seaweeds, and the structure of fucoidan consists mainly of polymers formed by branched polysaccharide sulfate esters with a l-fucose building block. Fucoidan has shown cytotoxic effects and induces apoptosis in MCF-7 cells. This apoptotic effect is triggered via the mitochondrial mediated pathway accompanied by activation of caspase-9 [126].

## 5. Conclusion

Apoptosis is mainly regulated by cell survival and the proliferative signal transduction pathway that is critically involved in human cancers. A balance of cell proliferation, survival and apoptosis normally maintains cellular homeostasis. However, apoptosis is a very complex process with numerous specific targets within each arm of the apoptotic pathways. Targeting cell survival and apoptosis signaling by marine compounds are useful for cancer therapeutics. Future research is required to identify the marine bioactive compounds-specific molecular mechanism for chemopreventive agents. The potential benefits of cancer chemoprevention from clinical trials and pre-clinical studies could be useful for cancer therapy.

## Figures and Tables

**Figure 3:**
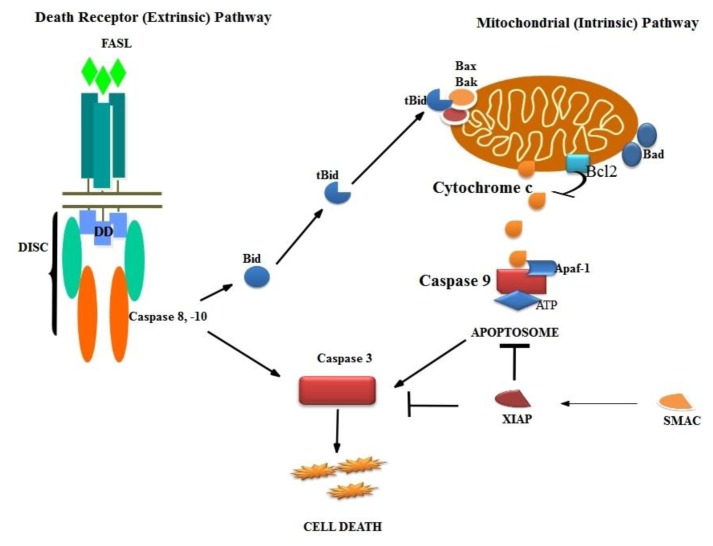
Pathway of apoptosis. Extrinsic cell death pathway is mediated by a TNF receptor superfamily, called the death receptors. Receptor-mediated cell death is initiated by the recruitment of adapter proteins, like FADD (Fas associated death domain), via the DD (death domain), which then bind to the death effector domain-containing caspase-8 or -10. Formation of this DISC (death inducing signaling complex) results in the activation of caspase-8/10, which then directly cleaves and activates the executioner caspase-3. Mitochondrial or intrinsic pathway, proapoptotic Bcl-2 family members, Bax and Bak, translocate to the mitochondria. The BH3-only protein Bid activates Bax and Bak oligomerisation form an oligomeric pore in the outer mitochondrial membrane. This results in the release of cytochrome c and other pro-apoptotic factors from the mitochondria to the cytosol. Cytochrome c triggers the assembly of the apoptosome (Apaf-1, caspase-9 and also the nucleotide adenosine tri phosphate (ATP) as a third component, binds and forms apoptosome). Subsequently, apoptosome activates caspase-3 and cell death. IAP (inhibitor of apoptosis protein) binds directly to caspases and inhibits their enzymatic activity. The inhibitory function of IAPs is controlled by the SMAC (Second mitochondria-derived activator of caspases).

**Table 1:** Anticancer compounds from marine environment.

No	Name of the Compound	Source of Organisms	Chemical class	Cancer Target	Reference
1	Arenamides A–C	Actinomycete (*Salinispora arenicola*)	Cyclohexa-depsipeptides	Human colon carcinoma cell line (HCT-116)	[107]
2	Heteronemin	Sponge (*Hyrtios sp*.)	Sesterterpene	Leukemia (K562 cells)	[108]
3	6-bromoisatin	Whelk (*Dicathais orbita*)	Indole derivative	Ovary, granulosa, Choriocarcinoma (OVCAR-3, KGN, Jar)	[109]
4	Tyrindoleninone	Whelk (*Dicathais orbita*)	Indole derivative	Ovary, granulosa, Choriocarcinoma (OVCAR-3, KGN, Jar)	[109]
5	Cryptosphaerolide	Ascomycete fungal strain CNL-523 (*Cryptosphaeria sp.*)	sesquiterpenoid	Human colon carcinoma cell line (HCT-116)	[110]
6	Makaluvamine A	sponge (*Zyzzya fuliginosa*)	pyrroloquinoline	Colon cancer (HCT-116 cells)	[111]
7	Ascididemin	Actinomycete (*Salinispora arenicola*)	Cyclohexa-depsipeptides	Human colon carcinoma cell line (HCT-116)	[112,113]
8	Lamellarin D	Prosobranch mollusc of the genus (*Lamellaria)*	Alkaloid	Leukemia	[114,115]
9	Spongistatin 1	Sponges (*Spirastrella spinispirulifera* and *Hyrtios erecta*)	macrocyclic lactone	Leukemia (Jurkat cells)	[116]
10	Streptochlorin	*Streptomyces sp*.	Methyl pyridine	Leukemia (U937 cells)	[117–119]
